# Visual Exploration of Financial Data with Incremental Domain Knowledge

**DOI:** 10.1111/cgf.14723

**Published:** 2022-11-28

**Authors:** Alessio Arleo, Christos Tsigkanos, Roger A. Leite, Schahram Dustdar, Silvia Miksch, Johannes Sorger

**Affiliations:** ^1^ TU Wien Vienna Austria; ^2^ Centre for Visual Analytics Science and Technology (CVAST) Vienna Austria; ^3^ Distributed Systems Group (DSG) Vienna Austria; ^4^ Complexity Science Hub Vienna Austria

**Keywords:** Visualization, Information Visualization, Visual Analytics, Visualization in Finance

## Abstract

Modelling the dynamics of a growing financial environment is a complex task that requires domain knowledge, expertise and access to heterogeneous information types. Such information can stem from several sources at different scales, complicating the task of forming a holistic impression of the financial landscape, especially in terms of the economical relationships between firms. Bringing this scattered information into a common context is, therefore, an essential step in the process of obtaining meaningful insights about the state of an economy. In this paper, we present *Sabrina 2.0*, a Visual Analytics (VA) approach for exploring financial data across different scales, from individual firms up to nation‐wide aggregate data. Our solution is coupled with a pipeline for the generation of firm‐to‐firm financial transaction networks, fusing information about individual firms with sector‐to‐sector transaction data and domain knowledge on macroscopic aspects of the economy. Each network can be created to have multiple instances to compare different scenarios. We collaborated with experts from finance and economy during the development of our VA solution, and evaluated our approach with seven domain experts across industry and academia through a qualitative insight‐based evaluation. The analysis shows how *Sabrina 2.0* enables the generation of insights, and how the incorporation of transaction models assists users in their exploration of a national economy.

## Graphical abstract

We present a system for the exploration and visual analysis of time‐varying financial data at multiple scales. We also describe a methodology to generate financial transaction models from real data, to explore in our system. If you want to know more, our paper is available as open access and source code can be downloaded from GitHub.

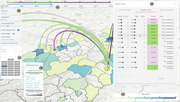

## 1 Introduction

The economy of a country is a complex ecosystem. The behaviour of companies and their relationships with each other cause effects that, combined, have repercussions on macroscopic, nation‐wide scale. Economists and financial analysts seek to understand and investigate these inner workings to devise profitable investment strategies or set up policies that have a stabilizing effect on markets and societal welfare. Unfortunately, there is generally no central source of information about the state of an economy. Such information has to be compiled from open (government) data, typically in the form of aggregate economic indicators [KCA*16], or monetary flows between industry sectors, and import/export records [TDL*15]. Other public and private sources include newspapers or corporate economic databases. Granular information about individual companies (or ‘firms') can be obtained through specific proprietary datasets cultivated by profit‐oriented research institutions [Sab]. However, the *transactions*, i.e. monetary flows between individual firms, are generally completely unavailable. Research on visualization of transactions typically accesses the data through collaborations with financial institutions or industry [KCA*16, CGK*07, DGL*18]. Such detailed monetary flows represent an essential piece of information when it comes to understanding transactional patterns, e.g. in which regions it is better to buy or sell specific goods or services, or which sectors (e.g. agriculture, manufacturing) are most and least proficient in which regions. Prior research attempts at simulating the relationships between the individual economic entities using agent‐based modelling techniques [PMT17]. These focus on obtaining aggregated data on a financial system after systemic shocks, disregarding the actions of the individual agents over time.

In this paper, we aim to provide analysts and researchers in finance and economy with access to the entire spectrum of available data in a national economy. This entails several challenges—besides the basic challenge of visualizing heterogeneous, spatio‐temporal and multi‐scale data, we deal with incomplete data: data on the level of individual firm‐to‐firm transactions. To fill these gaps, *Sabrina 2.0* integrates a pipeline for the inference of transaction models. These models are generated based on the publicly available higher level ground truth data, e.g. on monetary flows between industry sectors. The pipeline allows expert users to incorporate their domain knowledge within an inferred model, in order to obtain new insights or validate existing hypotheses and models about the relationships between companies and productive sectors in different regions. Within this context, we present *Sabrina 2.0*, a substantial extension of *Sabrina* [ATJ*19], a Visual Analytics (VA) approach designed to assist experts in two ways: first, by providing a unified visual interface for analysing financial data from heterogeneous sources in their geographical context at different level of aggregation, i.e. from regions and sectors up to precise locations and individual firms, and across time. Second, by enabling the exploration and comparison of multiple firm‐to‐firm transaction models observing their temporal evolution and providing both information on the individual transactions as well as aggregate statistics and metrics. Our contributions are:

- A design study in the context of economic data analysis: we derive a set of tasks for the domain, obtained through expert interviews and previous research experience in the field [ATJ*19]. We provide and discuss the results of an informative qualitative user study with seven domain experts, formulating lessons learned and future work.
- We crystallize our findings in a VA prototype, *Sabrina 2.0*, that takes full advantage of our pipeline for the generation of transaction networks [TASD19] enabling the exploration of transaction models with arbitrary constraint combinations.

## 2 Related Work

Financial systems are complex and hard to predict. However, the increased availability of financial data provides interesting and exciting research opportunities for the VA community [Teg99, DML14, KCA*16, FLVW16]. With the rise of big data, the unprecedented amount of real‐time financial information showed the limitations of traditional line charts, candlesticks and Excel‐based data exploration approaches. Ko *et al.* [KCA*16] survey several VA approaches for financial data, mostly stocks and fund trends, highlighting how these approaches suffer from limited scalability, overplotting and lack of possible interactions for domain experts especially when analysing multivariate and/or rapidly fluctuating data (e.g. price, availability, volume) coming from different sectors. While existing approaches (e.g. line charts) still provide powerful aggregation methods that reduce overplotting and are still widely used in the domain, in some cases, such loss of information might not be desirable, as in the analysis of spatio‐temporal data (e.g. comparison of sales or transactions).

**Multiscale geospatial visualization**. While geospatial information is typically presented in the context of a map, this approach is not very common in the financial domain [KCA*16]. Among those rare cases is *MarketAnalyzer* [KMJE12], a VA system for business analysis oriented to competitive intelligence in the retail sector using spatio‐temporal, multivariate point‐of‐sale data. This multiple coordinated view approach provides a geographical context to the visualization of buying/selling trends, supporting the visual analysis of spatio‐temporal patterns on a regional scale. This combination of views proved to be effective in tracking the popularity of products and evaluating possible investment opportunities. In contrast to our approach, the system is oriented towards business intelligence for market analysis and can evidence trends in sales in the local and regional scale, which is a narrower scope than our proposed system, which designed to uncover economy tendencies on different scales and across multiple sectors. Moreover, the geographical information only plays a secondary role. Mirel *et al.* [MGBC07] propose a visualization system for multivariate data about the international trading of commodities at a national and international scale. The visualization is embedded in a three‐dimensional (3D) environment, in which every aspect of the data is placed on different linked views and displayed on different planes of the virtual desktop (i.e. in a ‘walls and floor’ metaphor). The geographic information is shown in one of these planes as a two‐dimensional (2D) map, with 3D bar charts placed on top encoding econometric indicators. The paper presents a case study on the Caribbean basin and their protein (e.g. beef) consumption, but only few details about the scalability and the effectiveness of the approach are available. Khuu and Chan [KC19] investigate the potential of immersive technology for the 3D visualization of spatio‐temporal financial data. The paper proposes a 3D view of the Australian energy market on a map of the continent, using graphical assets to represent, e.g. the demand and average price of electricity. They argue that such abstraction presents several advantages, including showing more data than a 2D visualization, easing the process for discovering possible outliers; potential advantages of 3D exploration for economic data are also discussed. Schroeder *et al.* [SAHCV20] also investigate the use of mixed reality displays for the visualization of financial portfolios. In our approach, we considered these findings when choosing a 3D environment over a flat 2D visualization.

**Visualization of transactions networks**. Investigation of the individual relationships between fiscal entities is an established technique for economic forecasting [PMT17], understanding firms’ buying/selling practices [KSH*99] or to unveil suspicious or fraudulent behaviour [Her16, CGK*07, DGL*18, LGM*18]. Chang *et al.* [CGK*07] present *WireVis*, a coordinated multiple views VA approach to identify specific keywords into bank wire transactions and support the discovery of suspicious transfers. Its views include a heatmap that groups accounts and displays the keyword frequency per group, a node‐link view representing the relationships between keywords, and a customized line chart for analysis of wire activity over time. Didimo *et al.* [DGL*18] describe *TaxNet*, a VA approach for tax evasion discovery. The system presents a visual query language for describing suspicious transaction patterns that are stored in a graph database. Visualization is based on a node‐link approach whose nodes are the fiscal entities and the links represent the transactions. Tekušová *et al*. [TK08] project information about shareholder networks into a directed weighted graph to reveal patterns and support the visual analysis of cash flows. The visualization is based on a node‐link view, where each node represents a company connected through edges to their shareholders. Extra information about the firms is shown on demand through pop‐up windows. Given a specific user‐selected firm, the system supports the identification of its common shareholders and controlled companies. Leite *et al.* [LAS*20] present a VA approach for the exploration of cash flows between productive sectors in Austria. This information is encoded as an adjacency matrix on a national level. The tool allows the specification of ‘profiles’, i.e. a selection of sectors within a specific cash‐flow range, where relationships are visualized in a node‐link view.

**Transactions as flow maps**. Transactions, as geo‐referenced movements of money, can be visualized as origin–destination (OD) flow maps. Jenny *et al.* [JSM*18] identify design principles to foster readability and avoid visual clutter in OD flow maps. Boyandin *et al.* [BBBL11] present a technique for the exploration of temporal OD data. The origin and the destination of the flows are shown on two separate map views; a heat map in the middle connects the flows and displays their trend over time. Yang *et al.* [YDJ*18] investigate the exploration of OD maps in immersive environments.

**Our contribution**. From our analysis of related work, we understood how work on transactions heavily relied on collaborators from industry, who would provide data that would not be accessible otherwise. Despite the existence of modelling techniques that could simulate or infer the relationships between fiscal entities, little to no work has been done in the reconstruction of transaction models in a VA context. Moreover, we found the usage of visualization of financial data in a geographical context to be under‐investigated, which could be beneficial in identifying under‐/out‐performing regions, and in obtaining insights due to their geographical context and surrounding industrial environment [KMJE12]. With this paper, we aim at filling this gap in literature, providing a system that enables the visual analysis of heterogeneous geo‐referenced financial data, and the exploration of the relationships existing between productive sectors and firms and their evolution over time. Our system is designed to support researchers and policy makers in exploring and examining local and regional financial environments, in order to gain insights, make decisions and investigate resilience and the effects of shocks.

## 3 Design Principles of *Sabrina 2.0*

To design *Sabrina 2.0*, we followed the nested model approach by Munzner [Mun09]. We also took advantage of the ‘Design Triangle’ by Miksch and Aigner [MA14] to identify: (i) the target audience (the *Users*), (ii) the data model (the *Data*) and (iii) the tasks the system is designed to achieve (the *Tasks*). To build a robust categorization of the problem domain [Mun09], we conducted several intermediate evaluations with experts during the different stages of development. At the beginning of our project, we conducted guided interviews with two researchers that are experts in model building for financial data. Their feedback and guidance were condensed in a preliminary version of this system [ATJ*19]. The feedback to our initial approach and the collected insights and ideas were the basis for this work.

**Users**. *Sabrina 2.0* targets experts in finance and economy, such as financial data analysis and economists both in research and industry. Users from industry and government agencies assess the development of national and international markets and analyse the financial performance of individual firms as well as their impact on their geographical and industrial environment. They also monitor the effect of policies on the financial system. Target users are proficient in statistical data analysis but do not have to be experienced in model building and visual analysis. Experts from academia are interested in simulating and investigating the effects of shocks on economic models in order to quantify the resilience of financial systems and the effect of policy changes.

**Data**. We observe that information within the financial domain conforms to two major categories, which we refer to as *domain information* and *domain knowledge*. **Domain information** concerns structured data (tables, charts, trends, *etc*.), coming from either public or proprietary sources, encompassing information regarding specific firms or economic indicators about the financial performance of regions and sectors. We categorize these data as *‘micro‐'* and *‘macro‐data'*, respectively. An example of micro data is the individual companies’ balance sheets (usually publicly available). An example of publicly available macro data are *IO Tables* [TR10] that depict the interdependencies between sectors of a national economy, described in terms of financial transactions between industry sectors, including detailed records about import and export [TDL*15]. **Domain Knowledge** refers to unstructured information stemming from financial experts’ insights and experience, including expected sector growth, supply chain knowledge, experience in trading goods and commodities in specific geographical contexts.

*Sabrina 2.0* internal data model is designed to encompass both, domain information and knowledge, in the form of a weighted dynamic network. A network, or graph, is a data structure composed by a set of entities (nodes) and a set of relationships (edges) between them. Both nodes and edges bear numerical and text attributes and can change over time. In *Sabrina 2.0* data model, the fiscal entities represent the nodes of the graph. Nodes are then clustered together depending on the level of aggregation, from the individual firms (at the local scale), to a single region, up to the whole country. When clustered, individual node attributes are substituted with aggregate values. The financial transactions (i.e. the movements of money) between firms, entities, sectors and regions are represented as weighted edges. Our flexible organization of data can incorporate both domain information and knowledge, and can be customized to add new types of fiscal entities (or groupings thereof, such as holdings).

In the absence of measured or reported transaction data (a frequent circumstance in the domain, see Section 2), the transaction edges of such a network can be provided by an econometric ‘guesstimation’: that is an estimation made by guesswork or conjecture [WA08, TASD19], and such estimation is the first step of any economic empirical research [Cha02]. The common denominator of those heterogeneous information sources is the financial behaviour of individual firms within an economy, i.e. *how firms transact* with each other. In the context of this paper, we adopt an econometric modelling technique based on the definition of *constraints* generated from the available domain information and knowledge [TASD19]. These constraints are then used to generate a set of transactions, which we refer to as **transaction model** in the context of this paper. The transactions in the model inherently respect the given constraints by construction. The domain knowledge used to define these constraints, however, may either come from absolute truths, or from insights that an expert possesses and thus may be of varying quality, or even be conflicting with raw data. To mitigate this, our modelling technique also supports the creation of *incremental* models. These feature a set of ‘core’ constraints and multiple sets of cumulative constraints coming from different manifestations of domain knowledge. Each combination will generate a different transaction model: during the visual analysis, the additional constraints sets may be included (or excluded) within the analysis workflow, resulting in changes on the visualized transaction network. As such, experts can evaluate and experiment with different hypotheses and manifestations of domain knowledge. We describe the process behind the generation of the transaction models in Section 4.

**Tasks**. With consideration of the profile of the two categories of users which are part of our target audience (see **Users** paragraph) and the data *Sabrina 2.0* is required to process (see **Data** paragraph), our system must provide a VA environment where analysts can explore the financial and industrial environments at different scales, from individual firms to the financial and industrial environment of larger regions. Understanding the underlying web of relationships between firms is also crucial to assess the resilience of the local economy, and predict the effects of financial policies to be applied in a specific regional context. This last requirement also involves the possibility of testing hypotheses over the available data, comparing the effects of different assumptions when building financial models.

*Sabrina 2.0* was developed in close cooperation with domain experts, who were queried through the entirety of the development process, presenting them with intermediate prototypes in which we systematically revised our set of tasks and polished our visualization design based on their feedback. Our final set of tasks is the following:

**T1: National economy overview**. This represents the beginning of any top‐down exploration. At this stage, users are interested in overviewing the status of the economy at a large (national) scale: this includes browsing the geographical distribution of firms and productive sectors over the country, as well as aggregated financial metrics in reference to the national administrative divisions (e.g. wealthiest provinces, regional firm density).

**T2: National to local economy drill‐down**. Once an overview of the national economy has been obtained, the researcher would be interested in breaking down the aggregation from the national level into a smaller scale. The local economies of regions are influenced by the presence of prime materials (e.g., oil industry), orography (e.g., tourism) and climate (e.g., agriculture). Understanding the local distribution of productive sectors in small areas can provide valuable insights on the intervention of governments in the geographical context (e.g., presence of a high‐income industry in a low‐income area).

**T3: Local economy exploration**. The focus is shifted to the individual actors inside regions, provinces or arbitrarily small clusters of firms. The researcher is interested in particular and punctual information about a single firm or a very small group of them. This includes comparing their size and their contribution to predefined financial metrics.

**T4: Monitoring changes over time**. An essential question would be to understand if a policy change in a certain year, or the effect of shocks on the economy (e.g. the 2008 crisis) is reflected in the growth (or the demise) of companies and their corresponding financial turnover. The user investigates how the trends of financial metrics and the distribution of transactions between firms, regions and sectors evolve over time.

**T5: Investigation of relations between entities**. Firm interactions can be evaluated at different aggregation levels. However, with the available data, we can only evaluate the macroscopic effects of their interactions. At this point of the investigation, the user is interested in understanding the relationships between individual companies. This information would help painting the local economic context, i.e. by showing the firms' activity and suggesting how the largest firms influence and interact with the smaller ones in their vicinity.

**T6: Model comparison**. In the context of missing low‐level firm‐to‐firm transaction information, users have to resort to information sub‐sampled from higher level data via various network reconstruction techniques. The resulting models can reflect and incorporate different scenarios, depending on defined constraints. Further, models generated by different algorithms can vary in their accuracy and quality. These reasons make it necessary for the analyst to visually compare and evaluate different reconstruction models, in order to have a broader overview of the resulting model output space, inspect for conflicts and assess individual model quality.

## 4 From Domain Knowledge to Models

We categorize information within the financial domain into two major sources—*domain information* and *domain knowledge* (see Section 5, **Data** paragraph). In the econometric modelling technique used in this paper, such information must be encoded in an analysable form. Our approach is founded on the premise that one can merge different financial information sources to build a fine‐grained transaction network by incrementally introducing domain knowledge in the form of constraints over the available ground truth information. In the following, we give a high‐level presentation of the model generation process used in the scope of this paper. For a technical treatment, the interested reader is referred to the paper by Tsigkanos *et al.* [TASD19].

As the foundation, we treat information sources as logical statements. Those can represent elementary information, expressing simple facts that are known to be true. Such logical statements can, however, also have more complex forms, encoding relations between multiple allotted financial entities. This is highly useful when capturing domain knowledge that is often more aggregate or abstract. For example, information on the number of employees of a specific company may be explicitly expressed, while information about the aggregate financial performance of the sector that a company belongs to can be summarily and symbolically captured. A statement that a company c has five employees can be represented as the logical statement empl(c)=5. Domain knowledge may be encoded abstractly as quantitative statements. For example, knowledge that ‘companies in the Nikolsdorf area have between 1 and 5 employees’ is a logical statement that can be formulated as:

(1) area(c)==Nikolsdorf→1<empl(c)<=5.

Quantitative macro information, typically inherent in sector performance financial data, can be represented as behaviour of groups of companies—for instance,

(2) $$\sum_{i\in T_{s}}\sum_{j\in T_{t}}f\left(i,j\right)=10M$$

expresses that companies *i* within sector $T_{t}$ have collective flows to firms *j* in $T_{s}$ of 10M.

Logical statements obtained by following the process above correspond to different constraints over mathematically plausible models, and can be constructed programmatically. In practice, to build the firm‐to‐firm transaction model, constraints are encoded as first‐order logical formulae within satisfiability modulo theories (SMT [BT18]), using quantifiers (over finite sets) and integer linear arithmetic for specification. The result is a logical formula that describes domain information and domain knowledge together. A valuation that satisfies such a formula is a valid transaction model (i.e. it respects the defined logical statements) which is then included in the data model of *Sabrina 2.0*, as described in Section 3. In general, there can be many different graph variants satisfying the same constraints; the more constraints are specified, the better the transaction model approximates reality. We are concerned with the procedural aspects and implications of *creating* a model (‘How do we find an allocation?'), rather than qualitative issues (‘What makes a good allocation?') [CDE*06], the latter being out of our scope.

The generated network respects inherent constraints provided by the supplied domain knowledge and input data *by construction*. This reflects the design choice of adopting satisfiability to obtain transaction networks—we view other approaches, e.g. probabilistic, based on machine learning or agent‐based modelling as complementary to ours. We do not necessarily assume any concrete transaction data to be present, which would be necessary for data‐driven techniques, like machine learning. Additionally, transaction models obtained through other methods can be supplied to the pipeline as domain knowledge. Thus, the technique we adopt enables experts, who have independently developed a financial model—as is typical in the field—to complement and validate their work. The former concerns the case where an expert‐provided model is partial, i.e. not describing information for all firms. In this case, missing information is systematically guesstimated [TASD19]. The latter concerns the case where a provided model violates inherent data constraints of the domain, which are known to hold (a similar situation is described in the case study in Section 6.2).

## 5 Design and Implementation

In this section, we first give an overview of *Sabrina 2.0* and subsequently discuss the details of its components, indicating their most relevant aspects and design rationale in respect to the defined tasks and requirements.

**Figure 1:**
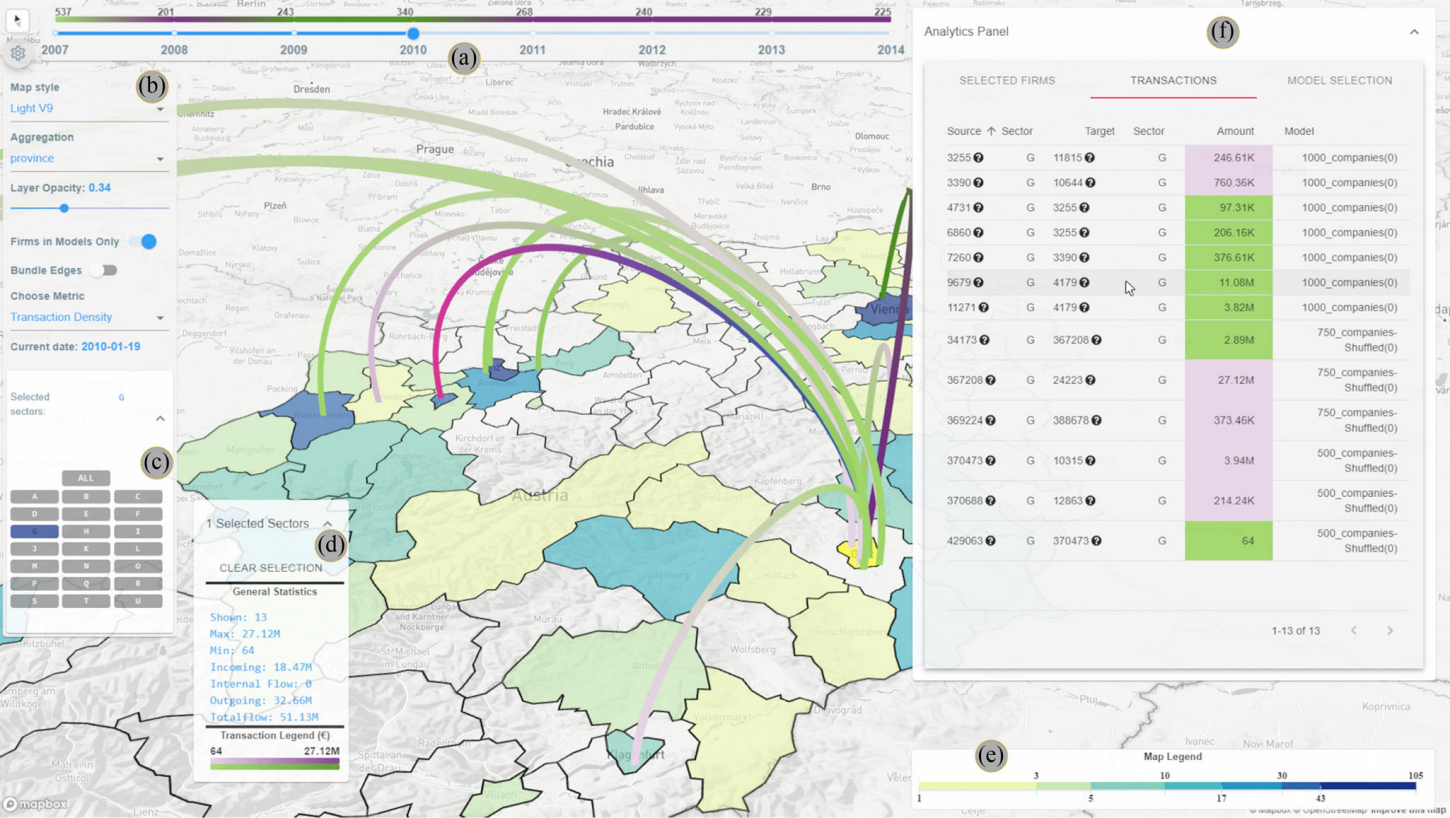
A full view of the system, as found in Case Study II (see Section 6.2): (A) the timeline, (B) the configuration panel, (C) the sector selection panel, (D) the details box, (E) the map colour legend, (F) the transactions table. In the transactions table, the mouse pointer is hovering over one transaction, highlighted in blue/magenta on the map.

**Figure 2:**
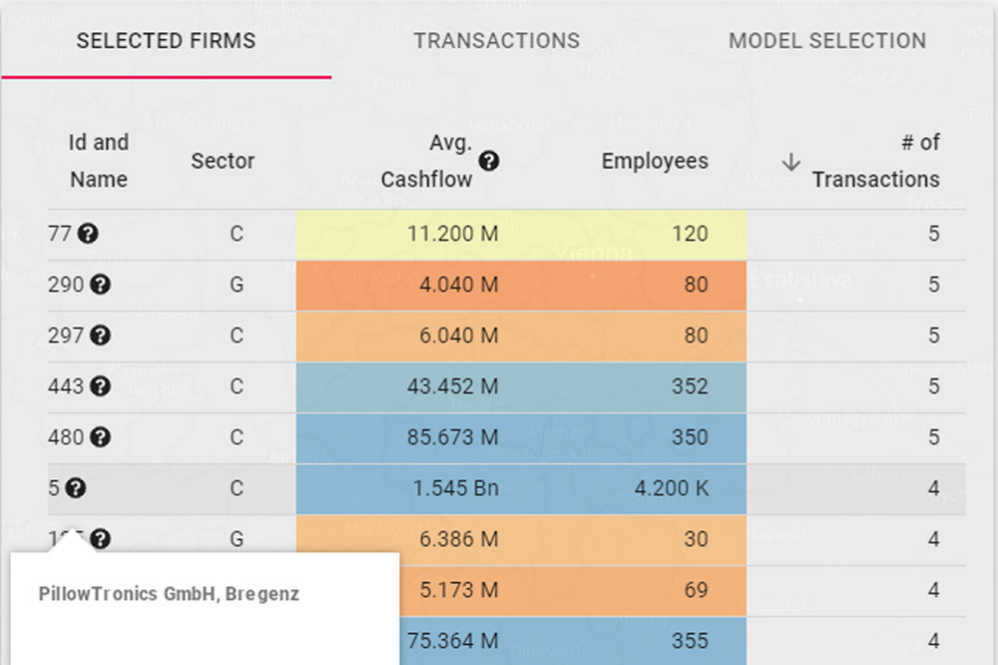
The firms tab. By clicking on the question mark, it is possible to access detailed information on the selected firm. The last column changes to show the specific contribution of the firm to the selected metric (see Section 5.1).

**Figure 3:**
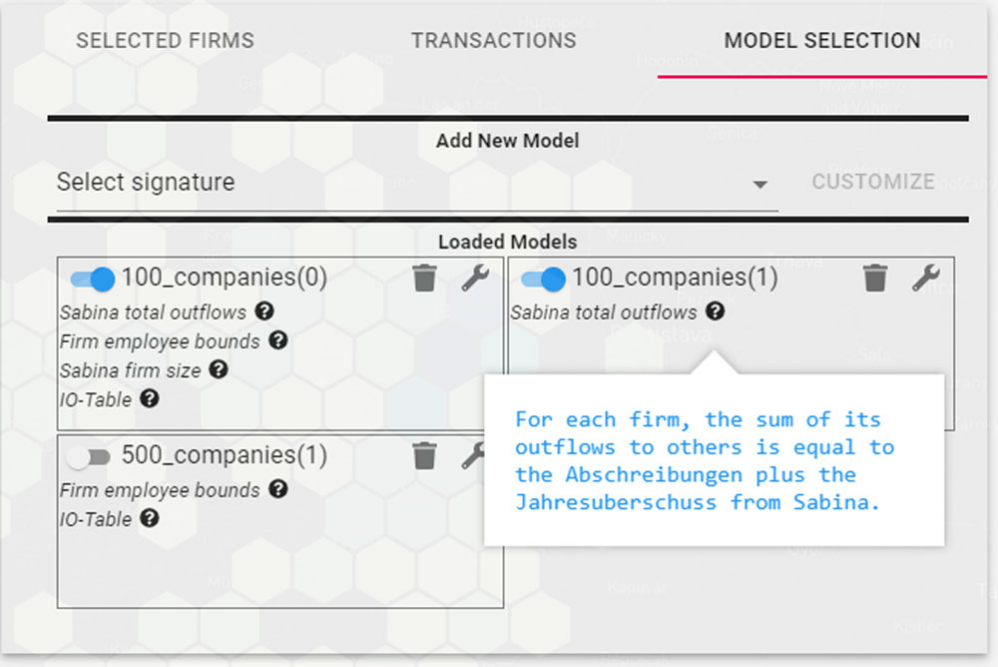
The model selection tab. The transactions from each model can be independently shown or hidden from the visualization. Active constrains are listed below the model name. Hovering on the question mark displays the constraint description.

The components of *Sabrina 2.0* are structured into coordinated multiple views, following an overview + details‐on‐demand approach (**T1‐3**). A full view of our system is available in Figure 1. The two main views are the **Map** view and the **Analytic Tabs** (Figures 1‐F, 2 and 3). The other notable components are the Timeline Slider (Figure 1‐A), and the Configuration panel (Figure 1‐B) that enables tweaking the visualization settings.

### 5.1 Map view

The map view's main purpose is to display the network data model (see Section 3, paragraph **Data**), comprised of firms and their transactions, within its geographical context. The base 2D map can be tilted, zoomed and panned. The geographical reference is an essential factor in the types of analyses that our experts conduct and supports the design tasks (see Section 3, paragraph **Tasks**), as indicated in the following.

**Visualizing firms**: On an abstract level, the data on which our system is operating consist of a dynamic network where nodes are the individual companies and the financial transactions between them are represented as weighted edges, encoding the transaction value from the loaded inferred models (**T5, T6**). Each firm is described by several attributes: first, general information, such as the address (which we geocode into coordinates), the name and the productive sector. Second, the yearly gains and losses, which provide information about its financial performance and acting as ‘ground truth’ for the inferred transaction models (**T5, T6**). Finally, we include the number of employees as an indication of the size of a company.

**Figure 4:**
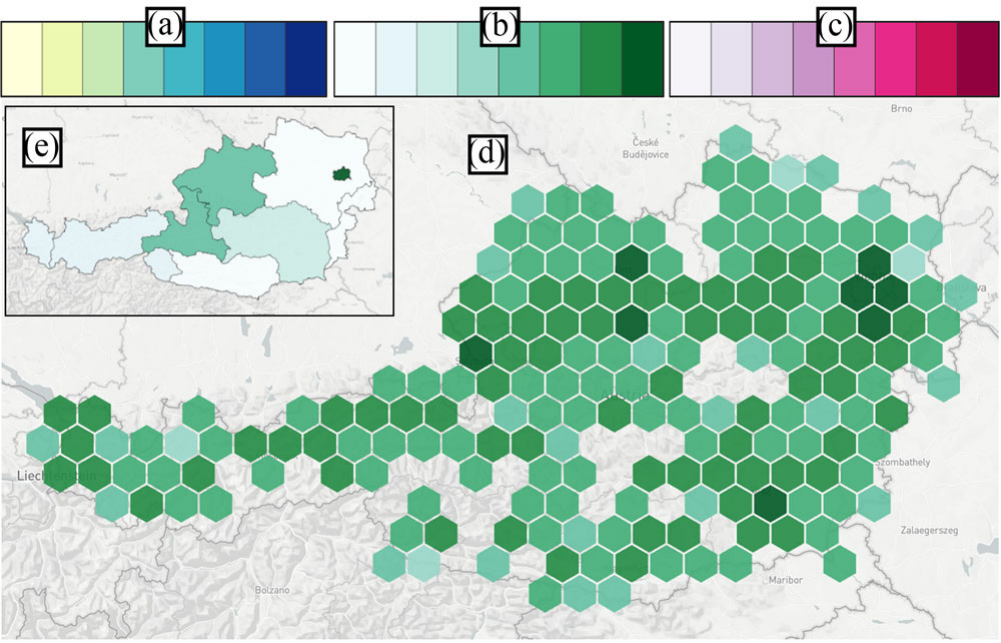
The colour schemes used to display the system metrics (top) and a snapshot of the hexagonal binning on the map (bottom). The colour schemes are used to encode density (A), gains (B) and losses (C). On the map, the selected metric represents the summary of firm gains (D). As a reference, the same metric has been applied to a region‐based firm aggregation in the window in the top left corner of the figure (E).

While the geo‐spatial context of the presented financial data is important, rendering each firm at their given coordinates results in severe over‐plotting and thus clutter in densely populated areas. Therefore, we aggregate firms, offering two approaches: based on proximity (see Figure 4‐D) or according to the administrative regions of a country (i.e. political regions, see Figure 4‐E). In the former case, we apply a hexagonal clustering (or ‘binning') on geo‐spatial firm locations. When compared to the commonly used square (or fishnet) binning [BOB07], the use of hexagons has several advantages that benefit the aim of our approach. Besides the increased visual appeal [CLNL87, Car90], they are less distracting than fishnets [COW92], due to human sensitivity to vertical and horizontal lines [CPMP98]. Hexagons also help convey spatial structures, and provide a clear representation of neighbouring regions (**T2**, **T3**, **T5**) [COW92]. In *Sabrina 2.0*, the hexagons’ diameter can be changed using the configuration panel (see Figure 1‐B) for a more fine or coarse representation of the underlying data. The second aggregation method allows users to inspect one or more administrative regions (**T2**). Firms will be clustered depending on the region/province they belong to. The opacity of the firms layer can be changed, to show the underlying geographical information such as the position of major cities and natural landmarks.

The choice of a regular tessellation over a non‐equal area projection (i.e. the underlying Mercator map projection) has potential hazards that must be acknowledged prior to using the system. First, while regular binnings are particularly appropriate for aggregation of point‐based measurements where it is important to maintain the measured value close to its location [BSF17], the reader might not compensate for the distortion introduced by the map projection (i.e. same spaced polygons in the tessellation have different surface areas on the sphere). Second, any map using spatial binning is affected by the modifiable areal unit problem (MUAP) [BSF17]. The problem refers to the representation of data whose values are affected by the shape and size of the geometry spatial units used: in our case, the shape of the hexagons or the regional and district boundaries. These artificially partition the data, and therefore, changing the hexagons’ size or switching administrative grouping will change the calculated summaries and, therefore, impact the interpretation of the results. Comparison tasks across multiple maps are the most affected, since if the individual bins are not of the same size and centred in the same position in all maps, they would not be directly comparable [BSF17]. While *Sabrina 2.0* offers a control to explicitly set the size of the hexagons, their centre point is calculated automatically and is not modifiable: however, comparison across multiple maps is not comprised in our system design tasks.

**Metrics and colour legends**: It is possible to compute several indicators, i.e. **metrics**, as aggregates over any group of firms (**T1**, **T2**). *Sabrina 2.0* provides six metrics: (i) the number of firms in the group, the aggregate balance sheet revenue for (ii) profitable and (iii) non‐profitable firms (i.e. firms with a positive/negative balance sheet that year), (iv) the *number* of incoming/outgoing transactions and the overall transaction cash (v) in‐ and (vi) outflow. Each metric is encoded with a different colour scale. The colour scales are discrete (with six classes), sequential and colourblind friendly [HB03], going from lighter hues to darker as the encoded value increases (see Figure 4). For metrics (i) and (iv), representing density values, we opted for a multi‐hue colour scale to highlight the difference between areas with high, mid and low firm density. Conversely, for the other metrics, we used a single‐hue colour scale. For metrics (ii) and (v), we use a light green hue, recalling the colour of a positive balance, i.e. an inflow of money. Similarly, for the remaining metrics (iii) and (vi), we use shades of pink to recall a negative balance.

Selecting a metric is a global setting that affects several components of the system. First, the metric is used to colour the hexagons/regions on the map, depending on the logarithm of their metric score. We employ a logarithmic scale since the metric scores might be dishomogeneous (e.g. densely populated areas vs. rural areas). While slightly harder to interpret for untrained eyes, logarithmic scales are common in the financial domain. The legend reference and the computed interval bounds are displayed in the **Map legend** (see Figure 1‐E). We arranged the tick values alternatively on top and bottom of the legend to reduce overlaps with larger numbers. Finally, we use the same approach to colour the sector selection area in the configuration panel (Figure 1‐C). Here, the metric is computed for all the firms belonging to each productive sector and the button is coloured according to its score following the same procedure as above. This gives the user information on the distribution of the metric value across all sectors (**T1**). The system is designed to easily enable the extension of the list of supplied metrics.

**Visualizing transactions**: The in‐ and out‐going transactions of the firms within selected hexagons/regions are visualized as a 3D ‘Flow map' [JSM*18], as displayed in Figure 1 (**T5**). 3D flow maps use the third dimension to disentangle the flows and reduce crossings [YDJ*18, VFAA17]. The trade‐off is perspective distortion. However, the view angle can be dynamically adjusted to alleviate the issue. Links are drawn as arcs that are bent upwards in respect to the map plane—resulting in reduced occlusion between the hexagon bins of companies on the map and the transaction edges between them. We set the arc height proportional to the distance of its source and target points, to vertically separate the crossings. The colour encodes the direction of the transaction and the brightness its amount. Direction in flow maps is usually encoded using arrows [JSM*18], but colour is also used when such a solution is impractical [YDJ*18]. We colour the edge in purple close to the transaction source to indicate a ‘loss'/out‐flowing money and in light green close to the target, representing a ‘gain'/in‐flow. In this way, regions with a high number of outgoing transactions (i.e. sellers) will be surrounded by green, and buyers by purple (**T5**). Similarly, quantity would be best encoded in flow width [JSM*18]. However, we experienced heavy clipping between close arcs in our early design experiments, and, therefore, chose to use the brightness of the colour, following a continuous relative scale; an acceptable alternative in cartography [JSM*18, DWCM18].

**Figure 5:**
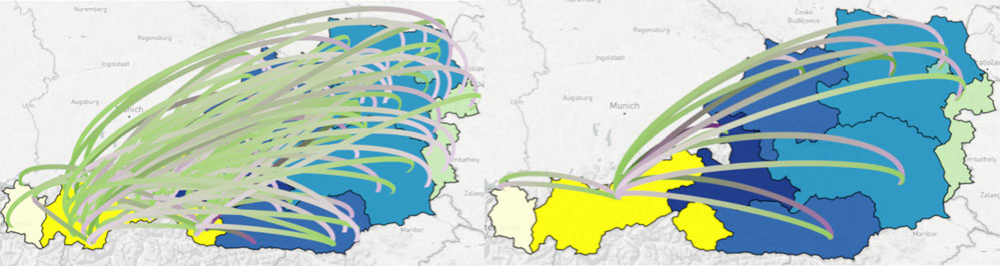
Visualization of the in‐ and out‐going transactions of a region (Tyrol, Austria) without (left) and with aggregation (right).

**Arc aggregation**. Arcs are projected at the coordinates of their respective source and target firms. When large models are used, the increased number of displayed transaction arcs can cause a high amount of clutter. While filtering can alleviate the issue to a certain extent, we give users the option to aggregate transaction edges that share the same origin and destination region/hexagon. When arc aggregation is enabled (by default), edge values are summed up, resulting in a maximum of two edges, for each pair of hexagons/regions, i.e. one per transaction direction. Moreover, transactions that have both source and target in the same region/hexagon will not be shown on the map, but will still be marked on the Transaction Table with a small circle icon (see also Section 5.3, **Transactions Table** paragraph). Aggregation greatly reduces clutter and allows for a quick overview of the flows between different areas (**T2**), see Figure 5. In cases where a higher granularity is required for a proper exploration, e.g. when zooming into small areas (**T3**), aggregation can be deactivated thus showing all transactions available within the active models in the selected areas, including the ones previously hidden. Alternatively to arc aggregation, branching flows, i.e. a bundle of flows with same origin or destination is subsequently branched, could be used to convey transactions. However, this makes the individual transactions harder to follow, and, when applied to geographical contexts, tends to create misleading artefacts [AA13] (e.g. non‐existent arterial roads). Moreover, arc bundling is a much more computational‐intensive task than aggregation, and therefore less scalable for an interactive application.

**Figure 6:**
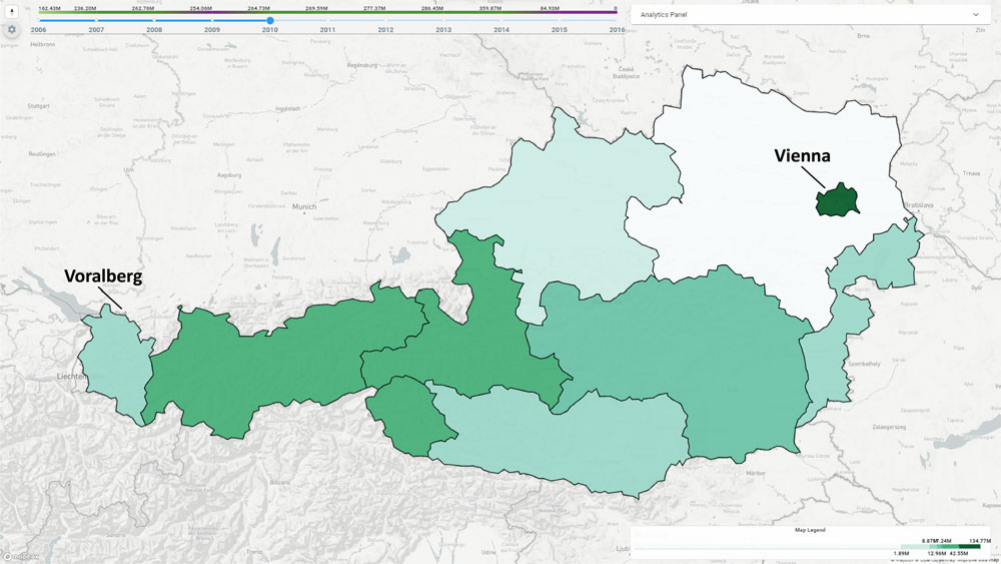
Distribution of tourism firms cash inflow, aggregated per administrative region, as discussed in Section 6.1. Colour scheme follows the one shown in Figure 4. The Vienna region shows a much higher financial turnover.

**Arc tilting**. When two or more transaction arcs share the same source and target coordinates, their respective arcs on the map might overlap, making it impossible to tell which is which. For each pair of firms/areas sharing more than one transaction, we place the corresponding arcs on each side of the imaginary line connecting their coordinates. If we consider 0° as the plane intersecting the map, we uniformly arrange the *incoming* transactions up to an angle of 90°; similarly, *outgoing* transaction arcs are distributed between 0° and $-90^{\circ }$ angles (see Figures 5 and 6). This way, it is possible to discriminate in‐ and out‐going arcs at a glance. The generous spacing between them is designed to make the transactions easier to track.

### 5.2 Interacting with firms and transactions

**Details on demand**. When clicking on a hexagon/region, the corresponding details of the firms belonging to the spatial region will be displayed in the firms table (see Section 5.3 and Figure 2), and the corresponding transaction arcs are rendered on top of the map (**T3**, **T5**). In the bottom left corner of the screen, a panel displays transaction statistics (i.e. % of in/outgoing funds, overall flow) and the transaction flow colour legend (see Figure1‐D).

When hovering over the map, a tooltip appears, informing the user about the computed metric value of the firms in that area and the economic sector distribution (**T2**). If other geographical information is included in the dataset (e.g. the name of the region or province) it will be displayed as well. When moving the mouse over a transaction arc, the tooltip shows the number of aggregated transactions and the sum of their values, if arc aggregation is active. Otherwise, the identifier, the sector and the name (where available) of the source/target companies generating the transaction are displayed. In either case, the transaction amount is reported as well.

**Filtering**. Firms can be filtered in two ways. First, by using the sector selection panel (see Figure 1‐C). By clicking on the sector icons, all companies belonging to the respective sectors can be excluded from the visualization and the metric computation. Since a transaction model might not incorporate all firms in the micro data, the second filtering option allows the user to hide the firms that are not represented in any of the currently active models (i.e. firms that do not receive or issue any transactions in any active model).

**Navigating time**. To investigate the temporal development of individual metrics, time can be navigated by interacting with the slider at the top of the map (Figure 1‐A). When the time step is changed, the active displayed firm set is updated, adding newly founded companies and removing the ones that ceased to exist, e.g. due to bankruptcy (**T4**). All aggregated measures are updated accordingly. To guide the user towards potentially interesting time steps in the series, we display the temporal trend of the selected metric for all firms above the time slider (see Figure 1‐A). The change of colour indicates an inversion of the trend: a green hue indicates an increase over the last time step, purple encodes a decrease and grey shows that there were no changes.

### 5.3 Analytics tabs

This part of the interface gives detailed information on selected firms and displayed transactions in two separate tables (see Figures 2 and 1‐F, respectively). A third tab is used for model management (see Figure 3).

**Firms table**. In this table, we show additional information about firms that belong to the subset of the selected areas of the map (see Figure 2). For each firm, the table displays the database ID, its name (if available), the productive sector it belongs to, its average declared cashflow, the number of employees and its contribution to the currently selected metric (**T3**). The cashflow and employee columns also apply a colour coding to provide at a glance whether the company is considered to be small, medium or large according to the official definition by the Austrian Chamber of Commerce [KMU]. This classification depends on both the company earnings and the amount of personnel. We used a divergent colourblind‐friendly colour scale (orange for small, yellow for medium and blue for large). The colour legend appears when hovering on the header of the cashflow column.

**Transactions table**. In this table, we show specific information about currently visible transactions (see Figure 1‐F). The visualization changes depending on the status of the edge aggregation option. If enabled, we display data on each transaction aggregate. This information includes its size (i.e. how many transactions belong to it), and its aggregated transaction amount (i.e. the sum of all the individual flows). The other two columns, respectively, depict the productive sectors that spent and gained the most money from the transactions in the aggregate. When edge aggregation is disabled, the table provides the firm and sector names for the source and target of the transaction, the transaction amount and the model it belongs to (**T6**). In both modes, the cell displaying the amount is colour‐coded in pink when the transaction is outgoing from the selected areas on the map and green otherwise. Hovering with the mouse over a row highlights the corresponding arc on the map (see Figure 1). In cases where a transaction source and target belong to the same group of selected hexagons or regions, we speak of ‘internal’ transactions. In this case, the cell has no colour coding (the amount cannot be defined as ‘out‐’ or ‘in‐going’ from the group) and a small round icon is displayed close to the amount value in the transaction table. If arc aggregation is activated, internal transactions are shown in the table but no arcs are displayed on the map (since they would have the same source and target coordinates).

**Models selection**. The last tab contains the controls to manage model loading (see Figure 3). Once a model is loaded, it is possible to select the constraints that will determine which transaction network to display. After the constraint selection is updated, the loaded models are displayed in the tab, allowing users to show and hide them from the visualization, to change the active constraints, and to remove them altogether. Moreover, the currently active constraints are shown below each model for quick consultation.

### 5.4 Implementation and data formats

*Sabrina 2.0* is implemented in Javascript. The UI is built upon a React environment, using ‘Deck.gl' [Wan19], a Web‐GL‐based platform for the visualization of large data. *Sabrina 2.0* is a client‐side application, i.e. the data is loaded and processed directly in the browser. While this setup has a higher toll on local system resources, especially in terms of memory, the system is platform independent, and simple to setup and run. Geographical and financial data are imported from comma‐separated‐value (CSV) files. The models (both their definitions and the actual transactions) are stored in JSON files. The system was tested in Chrome (Version 83.0.4103.106, 64‐bit—official build), running on an i7‐8750H CPU with 16GB of RAM. The code, demo datasets, a link to a demo video and further information are available online [Git].

## 6 Case Studies

In order to illustrate potential workflows in *Sabrina 2.0*, we describe two case studies, carried out by one of our domain experts. We deployed our system to a researcher at the Complexity Science Hub Vienna, whose current work involves assessing the resilience of the national economy. Central aspects of this analysis involve the relationships between productive sectors at national and regional level, and the respective supply chains. The domain expert used our system in two scenarios that correspond to representative questions posed in their daily work. The system was prepared in the same way and loaded with the same data as in Section 7.1.

### 6.1 Case I: regions correlations

**Figure 7:**
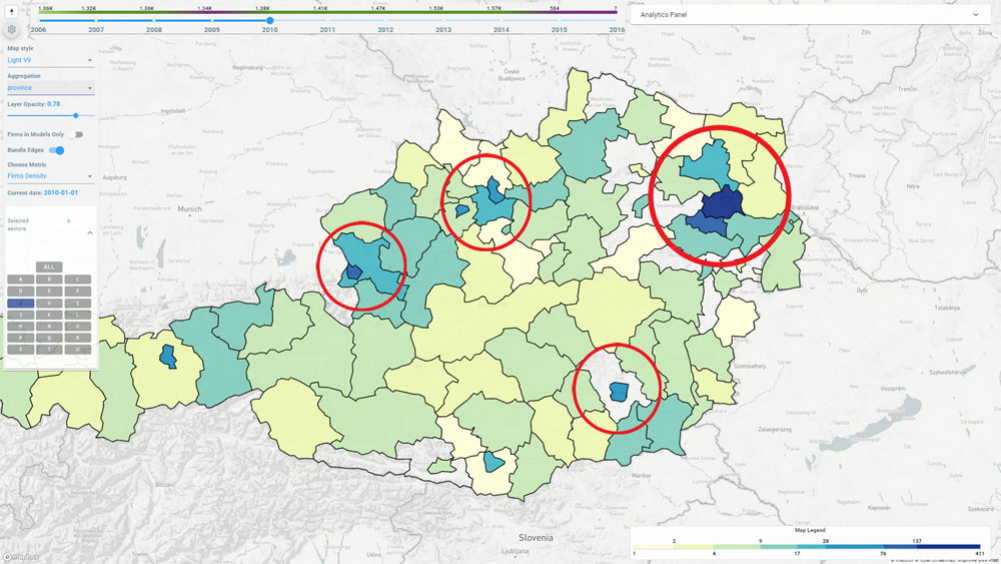
Car parts, repairs and manufacturing firm density in Austria in 2010, according to our data. The areas with a higher concentration of firms are highlighted in red. According to our case study, for a model to be considered as reliable, it should contain transactions between the firms in these areas related to vehicle repair and manufacturing (see Section 6.2).

The first question the user had to investigate through our system was: ‘If an economic shock occurred in Vienna, how vulnerable would the Vorarlberg region be?’. The shock in question was the impact of the Covid‐19 pandemic and the following restrictions put in place, with Voralberg being the westmost Austrian region and the farthest away from the capital. As our available data are dated from 2006 to 2015, the expert fixed the time to 2010, pretending that this shock would take place in the aftermath of the 2008 global financial crisis (**T4**). As first step, the user switched the firm aggregation to administrative regions and selected the two regions of interest to this case study (Vienna and Voralberg) and the ones that were immediately adjacent. By using the firms panel, the user could easily inspect the sector composition (**T1**, **T2**). The pandemic restrictions primarily affect the tourism sector, therefore, a region with a higher density of tourism‐related companies and economic activities would likely be more vulnerable. To assess this, the user switched the metric to ‘Gaining Firms Inflow’ and filtered out all firms except the ones belonging to the tourism sector to inspect and compare its overall financial performance in the two regions of interest (**T3**). The status of the visualization at this point can be seen in Figure 7: In terms of cashflow, the Viennese region has a much higher financial turnover than Voralberg, and therefore, could be more vulnerable in case of a shock. This high‐level analysis provides a first indicator of vulnerability.

At this point, to the expert focused on determining the specific relationships between the Viennese tourism firms and the companies based in Voralberg. With the pandemic closing restaurants and hotels in Vienna, it is safe to assume that the shock impact would spread through the transactions network between the companies of the two regions. Specifically, the firms in Voralberg which provided goods and services to the now closed activities in Vienna would experience reduced cash inflow. To investigate this, the expert chose three of the available models, namely two with 500 firms (one with randomly sampled firms) with all four constraints enabled and one with 200 random firms but only with the *IO‐Table* constraint enabled (see Section 7.1 for data and constraint description). As the expert was not familiar with our model building methodology, this first selection of models was made mostly out of curiosity, trying to assess the distribution of the transactions per firm and sector and the rationale and impact behind the model constraints. After investigating the available transaction arcs, only one of the loaded models (500 random firms) contained cash flows between (tourism) companies in Vienna and their suppliers in Voralberg (**T5, T6**). Specifically, these flows suggested that if tourism firms in Vienna were affected by a shock, following the outgoing transactions, the firms in Sector ‘J’ (Information and Communications Technology sector, according to Austrian economic activity classification) in Voralberg would be the most affected. With the available data, the expert concluded that the flows between vulnerable (tourism) companies in Vienna and their suppliers in Voralberg do not suggest a high co‐vulnerability due to their relative low value, especially when compared to the flows existing between other regions (see Figure 6). While the first conclusion concerning firm cash flows is a consequence of the available ground truth, this last one depends on the reliability of the loaded models, which brings us to the next scenario.

### 6.2 Case II: model comparison and verification

**Figure 8:**
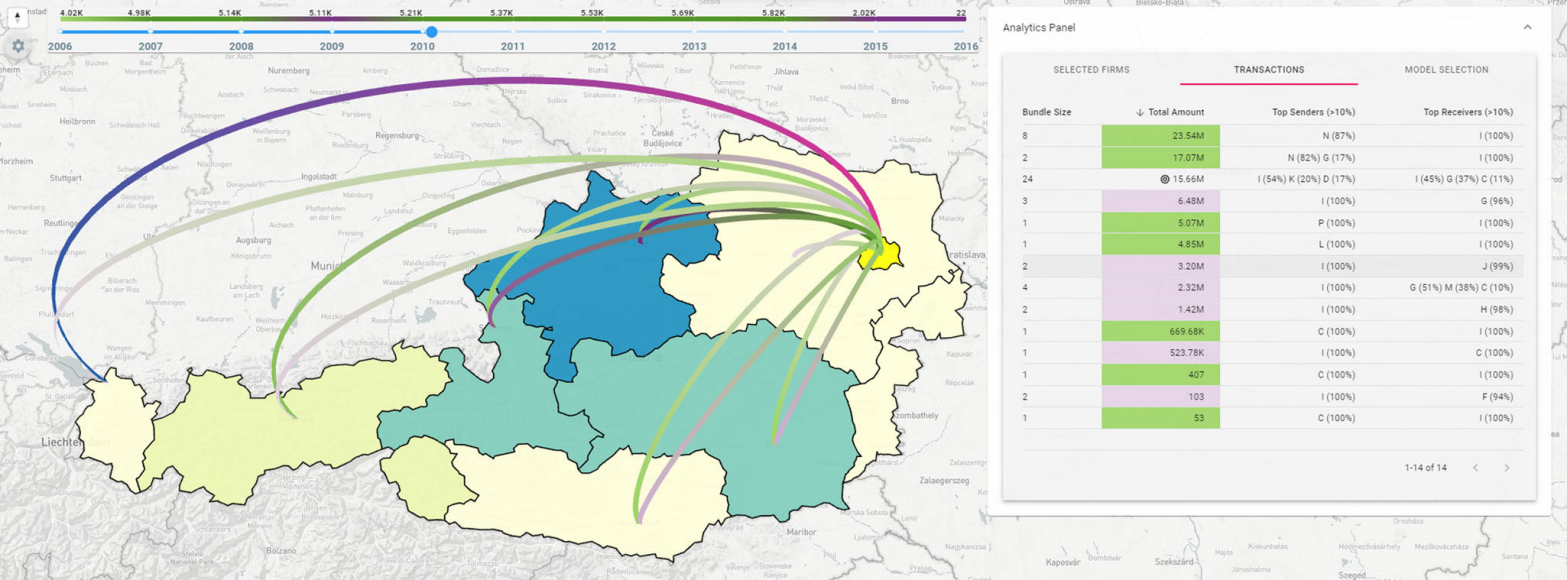
Closeup of the system status at the end of case study I (see Section 6.1). Among the transactions present in the model, the cumulative cashflow from Viennese to Voralberg firms (highlighted row in the table) is inferior to the one between Vienna and other regions, thus suggesting limited correlation.

The expert focused on the task of comparing and validating the different available models. In this scenario, the objective is to assess the robustness of the models in relation to the ground truth or, in the expert's own terms, do a ‘*sanity check*’ of the model. One possible indication of a model's reliability comes from the presence of known flows between companies, either coming from known sources or the expert's own domain knowledge. The user also wanted to compare the models loaded in Case Study I (see Section 6.1) with the other larger ones available, and therefore loaded the 750 and 1000 shuffled firms models into the system (both with all constraints enabled) along with the previous ones (**T6**). The focus of the exploration shifted towards automotive industry, as the expert explained that the presence of specific supply chain connections, i.e. car parts built in Lower Austria, that are then assembled in Upper Austria, are known and found in ‘*newspapers and even in the company's website*’. Therefore, they are the perfect candidates to perform a sanity check of one or mode models, and fit *Sabrina 2.0* workflow. Thanks to the geographical context given by the map, it was very easy to find the companies’ whereabouts, located in areas with higher density of automotive parts manufacturing firms (see Figure 8). Edge bundling was deactivated, in order to obtain a fine‐grained representation of the transactions, and all companies not represented in the model or not belonging to the trade of motor vehicle parts and accessories were filtered out (**T2**,**T3**). Only one of the loaded models (specifically, the one with 1000 companies) had the transaction the expert user was looking for: it is visible under the mouse cursor hovering over the Transactions tab and highlighted on the map in Figure 1 (**T6**). The expert could conclude that the formulae that generated that specific model were the most reliable in providing a realistic instance of the regional transaction network. Therefore, analysis and validation could continue on that model, possibly expanding it with other firms. The outcome of this analysis process could not fully validate the insights obtained in Case Study I, as the 500 random firms model did not present the transactions the user was looking for. However, that model features a few transactions in the neighbouring area that fulfil the analysis constraints (see bottom of the transactions tab in Figure 1). For this reason, the expert also observed that this could have been a consequence of the different number of firms in the model (500 vs. 1000) and differences in firm sampling.

This specific scenario also applies when external models, i.e. not computed using the pipeline described in Section 4, are loaded into the system for analysis and comparison. Further examples of *Sabrina 2.0* use cases can be found in the supplemental video.

## 7 Insight‐based Evaluation

In the scope of this paper, we focus on evaluating the aspects of *Sabrina 2.0* concerning the visualization and interaction design. For an evaluation of the model generation mechanism described in Section 4, we point the interested reader to the paper by Tsigkanos *et al.* [TASD19].

To validate *Sabrina 2.0*, we conducted an insight‐based evaluation [Nor06]. This method helps us determine what users can take away from the visualization, i.e. which insights users can gain on their own. The collection of usability data in conjunction with the coded insights would then allow us to validate our design goals. Insight‐based evaluation requires the participation of domain experts and an open‐ended think‐aloud protocol, discussed in this section. In the scope of this qualitative evaluation, we investigate whether *Sabrina 2.0* achieved the following goals, tied to the design tasks of our system:

- **G1**: *Sabrina 2.0* enables the creation of insights at every scale (national, regional and local) through a comprehensive overview and detail exploration of financial data (**T1**, **T2**, **T3**, **T4**);
- **G2**: By providing inferred transaction models, *Sabrina 2.0* enables the generation of new insights on firm behaviour and local economies (**T5**, **T6**);
- **G3**: *Sabrina 2.0* improves the current workflow of the experts, with little to no training needed.

### 7.1 Data setup and model generation

We used a version of the Sabina database [Sab] as micro data, which is a proprietary catalogue of all the Austrian registered companies and holds record of their presented balance sheets. To generate the inferred models with our pipeline (see Section 4), we first randomly sampled the micro data to create three groups of firms with increasing size: 200, 500 and 750. Then, we created another five groups of firms, with sizes 100, 200, 500, 750 and 1000, sampling the firms gradually from West to East: the ‘100 companies’ model would only contain firms from the western part of the country, the ‘200 companies’ model would include the original 100 and an additional 100 towards the east and so on. For each firm set, we computed a transaction model using the IO Tables for Austria as macro data. Moreover, each model comes with four extra constraints that can be activated from the model selection tab: (i) *Sabina Total Outflows*, where the total outflows for each firm are derived from the cash outflow figure in its balance sheets plus inflation; (ii) *Firm employee bounds*, where the personnel expenses are limited, simulating firms of up to 99 employees; (iii) *Sabina Firm Size*: the firm size depends only on its personnel expense (and not also on their final balance) and (iv) *IO‐Table*: the aggregate flows between firms are equal to the values from the IO Tables. These constraints were chosen to demonstrate the process of considering incremental new data in the creation of models (i and iv) and to simulate the use‐case of hypotheses verification (ii and iii). Considering that each combination of parameters yields a different model (see Section 4), we computed 16 transaction networks for each group of firms, for a total of 128 user‐selectable models, generated offline using the Z3 SMT solver [DMB08].

### 7.2 Participants

For choosing participants, we considered two factors: their relevance to the target domain [Nor06] and their prior knowledge of the Sabina dataset [Sab] and the Austrian economy. The candidate selection process resulted in a group of seven volunteering domain experts (five males and two females). Five of them (four males and one female) work in the Austrian Chamber of Commerce carrying out analytical work in policy making for companies, as well as quantitative data analysis for generating financial fact sheets. In their workflow, they mostly employ *Excel* and *R* for data analysis. For the visual representation of their data, they normally use static charts, plots and *‘everything related to Excel'*. Only one of them had prior knowledge of the Sabina dataset. The other two participants have an academic background: the first had long‐running experience with the Sabina data, which he had used in reconstructing the regional economy of Austria using agent‐based modelling (see Section 2). The last participant is an expert in visualization and had worked on financial fraud discovery through the analysis of bank transactions, with no prior knowledge of the data used during the evaluation. The experts were interviewed in three separate sessions over Skype; the two experts from academia were interviewed separately. None of the study participants had prior experience with the use of SMT solvers to build econometric models. One participant from academia had used agent‐based modelling in their studies on the Sabina dataset.

### 7.3 Procedure

We planned each session to last from 60 to 120 min (according to the number of participants), and recorded voice and screen during the entire session for post‐evaluation transcription. We divided each session into four fractions: first, we interviewed the participants to better understand the demographics of the experiment. We asked them about their daily workflow and tasks, the type of data that they investigate, the software and tools that they use to to handle the tasks and their experience with visualization. Afterwards, we presented *Sabrina 2.0*, and gave a tour of the features of the system, following a hypothetical use case. During this phase, participants were able to ask questions about the controls, visualizations and general inquiries about the underlying data.

The next phase, the core of each session, was an insight‐based evaluation using an open‐ended think aloud protocol, in which the users could explore the system without particular tasks or time constraints. For this phase, we gave users control over the system, by supplying them with access to the web application. During this phase, participants shared their screen with us. In the session with multiple participants, they shared the same screen in a collaborative fashion. We had a set of initial tasks to encourage exploration at every scale: e.g. identify and rank the Austrian regions by number of firms/cashflow, load at least one model and examine the transactions, identify—in a specific region—which firms or groups of firms had the most transactions. However, as all the study participants worked on the analysis of the Austrian economy, they already had their own questions and research goals which they pursued during the study sessions. We monitored the participants closely in this phase, answering any upcoming questions and eventually helping them if they asked for assistance. Our interventions, however, did not guide the users in using the system. We considered this phase concluded when the user(s) communicated to have found all of their answers in the given data. Finally, the session was concluded with a set of post‐evaluation questions and general feedback, in which the users were encouraged to tell us about their experience with the system and whether or not it would benefit their daily workflow.

### 7.4 Results

An insight is defined as an ‘*individual observation about the data by a participant*' [SND05]. We transcribed the session recordings and evaluated them to categorize and rank the different insights [Nor06]: We use this information, along with the answers to the feedback session, to draw a conclusion on whether or not our goals were met. Considering that the goals were formulated in direct correlation with the design tasks, they would also shed light on whether the system enables these tasks as well. Moreover, we also collected usability data and considerations for correlation with the insight results (see Section 8).

We categorize the insights as either *Exploration*‐ or *Model*‐related: the former includes observations that do not involve the use of models; the latter entails insights that could only be obtained by loading and inspecting at least one model. We further classify insights according to the scale they refer to, i.e. if they pertain either *national*, *regional* or *local* aspects of the economy. We choose this two‐dimensional categorization as it allows us to derive conclusions (a) on the impact of models on the overall number of insights (i.e. if the usage of models provides a more profound exploration of the underlying financial information), (b) on the capability of the system in providing information on the three different levels of detail and (c) on how the users interact with the system. Specifically, national level insights do not necessarily require extensive interaction with the system, while smaller‐scale (regional or local) insights, require more interaction with the system's features, such as loading models, selecting regions or hexagons, *etc*. Concerning *Exploration* insights, on a national level typical examples include the identification of the most prominent economic sector(s) or best performing firm(s) across the whole country. We consider an insight to be national when it concerns more than two (non‐adjacent) regions, or involves the presence/performance of a limited selection of sectors/firms that are scattered throughout the whole territory. A regional finding typically spans a single administrative region or a spatial subset of firms. An example is the density of productive sectors in a *single* province/region or in a group of firms within a group of adjacent hexagonal bins of comparable size. A local insight could concern details on single firms, e.g. the number of employees, or the characteristics of the financial and industrial environment of a very small area. We encode an insight as *Model*‐based when it was specifically obtained after observing transaction flows, such as newly found supply chains between more than one non‐adjacent region (national), and investment networks that support smaller economic environments (regional and local). We provide a set of example insights we recorded during the evaluation with the corresponding coding in Table 1. Finally, we categorize insights based on participant types: *academia* and *industry*, i.e. whether they work in research or in a company/government institution, respectively. With this categorization, we aim at understanding the differences in the usage of the system between these two categories of users. The summary of collected and categorized insights is displayed in Table 2.

**Table 1:** A selection of insights recorded during the evaluation as described in Section 7.4

	Exploration	Model
**National**	*‘There is a higher number of firms with losses after the 2008 crisis’*.	*‘The model* companies_500 *contains only transactions from ICT firms’*.
	*‘Agriculture firms are under‐represented is in this dataset (as they do not have to present balance sheets)’*.	*‘Model* 750_Shuffled *looks more realistic due to the increased presence of transactions from manufacturing firms’*.
**Regional**	*‘In 2013, Innsbruck tourism firms had half the losses of their Viennese counterparts'*.	*‘Model* 200_companies *targets firms in the Voralberg region, with most of its outgoing transactions directed eastbound’*.
	‘*Upper Austria has a higher concentration of firms related to vehicle production and construction equipment’*.	*‘In Model* 500_companies, *there is a peak of transactions going into firms in the Voralberg regions in the year 2008’*.
**Local**	*‘The top financial sector in Vienna is Professional, Scientific and Technical services’*.	*‘High flow of transactions from tourism related firms at the border with Slovenia’*.
	*‘There is an increased number of tourism firms at the border with the Alps’*.	*‘In the model* 500_companies, there is only one firm represented in Vienna’.

**Table 2:** The number of insights categorized by participant and scale. In brackets, we indicate the contribution of insights regarding models

	National	Regional	Local	**Total**
Academia	8 (3)	16 (4)	10 (4)	34 (11)
Industry	5 (1)	10 (6)	7 (5)	22 (12)
**Total**	13 (4)	26 (10)	17 (9)	56 (23)

**Figure 9:**
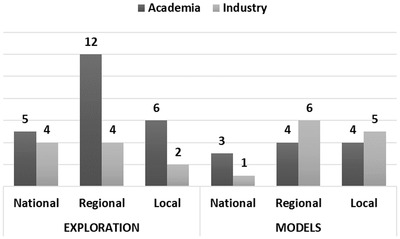
Insight distribution by scale. The number of insights is indicated on top of each bar.

**Overview**. We counted a grand total of **56** insights, with an average value of 8 per participant. The highest number of observations took place on the regional scale (26, i.e. 46.4%), followed by the local (17, i.e. 30.4%), and national scales (13, i.e. 23.2%). The contribution of model‐inspired insights was substantial, with 23 insights (41% of the total). The insight distribution varies between participant category and exploration type (see Figure 9). Subjects from academia had more insights during *Exploration*, with more observations on regional and local scale. Industry, on the other hand, achieved less insights in total, more densely distributed on the national/regional scale. For *Models*, the number of insights is even between the two categories, with a common trend towards regional local findings.

**Considerations about usage and user preference**. The insight rate and classification reflect how the users interacted with the system and with the transaction models. Participants from industry immediately aimed at checking specific areas/sectors likely to be of interest in their line of work. Most of their attention was focused on the distribution of firms in Vienna and the surrounding regions, both in terms of density and variety. Other than finding the most prominent sectors, they were also interested in specific ones, such as ICT, professional activities and education. Concerning the model generation feature, the participants asked many questions about how the transactions were generated, and the significance of the parameters each model had. When exploring the models, their focus was to understand the mutual financial relationships between the regions, based on the amount of transactions and the involved sectors, trying to find correlations in a similar way as we discussed in Section 6.1.

The participants from academia used *Sabrina 2.0* to evaluate the effects of the 2008 crisis on Austrian firms. They mostly used the cash in‐/out‐flow metrics (see Section 5.1) and firm density to assess the economic losses. While they were familiar with the dataset, they had rarely explored it with an explicit geographical reference. They easily found supply chains they were aware of from their work (e.g. ‘*…a south‐west trail of firms from Vienna*') and also pointed out missing information from the data. The majority of exploration was then focused on investigating temporal financial trends of specific regions from the available micro‐data, with limited interest in the models. As they also research methods to simulate transactions (i.e. agent‐based modelling, see the participant description in Section 7.2) they were more interested in exploring the dataset with this, for them, different approach, i.e. centred on the geographical facets.

It is possible to see the patterns we just described for both industry and academia participants in the sample insights reported in Table 1. In general, the hexagonal binning for the proximity aggregation of the firms was well‐received (‘*It feels like everything fits together*'); in fact, both participant groups preferred it over the region/province aggregation due to its higher resolution and granularity. The transaction (arc) aggregation feature was appreciated for the tidier overview it provided and was used for most of the exploration. Only in a few occasions, it was deactivated for investigating small groups of firms. Users further appreciated how the bundles’ aggregate information was displayed. Finally, the base map representation was deemed ‘interesting and pertinent’ to the domain by the participants from industry, basically confirming our design decision (‘*It's interesting to be able to understand whether something is happening east or west or if it is nationwide*').

**Study limitations**. It can be noticed that the average number of insights per user differs significantly between the two user categories. This difference also stems from how the evaluation was performed. Due to temporal constraints and the effects of the Covid‐19 pandemic, we could only conduct one session with the industry participants. Therefore, while the experts from academia were interviewed one by one, the industry session was conducted with all experts present at the same time. We opted for a group session in order to get more diverse feedback from these hard‐to‐get experts. In this session, users did not take turns, but rather used the system together, resulting in the lower number of insights per person. The post‐evaluation questions were asked individually.

### 7.5 Evaluating design goals

To evaluate **G1** (insights across economy scales), we focus on the distribution of insights, on the feedback, and on observations concerning how users explored the different scales. We observed similar patterns across the evaluation sessions: After understanding the national context and evaluating different metrics, users explored interesting areas or economic sectors in a top‐down fashion. We observed that often they were already aware of the region where to focus their smaller scale exploration: These were regions with large companies (e.g. *Red Bull* in the Salzburg area), areas of interest to their prior research or personal interest (hometown, touristic areas). The majority of insights were obtained at the regional level. We consider this a consequence of how users adjusted the visualization while transitioning to a local view (e.g. changing the diameter of the hexagonal binning). In the process, participants began to make connections between progressively smaller clusters, evaluating the productive sectors’ distribution and density. Insights at the local level were less numerous also due to the limitations of the data: As the zoom level increased, companies appeared sparsely distributed and some key firms were missing. We observed that at every level, the first insight of each participant concerned a piece of information that they expected but that was not present in the data, such as a missing company/productive sector in an area. This encouraged users to enable and explore models with different constraint combinations to substitute the missing information. The participants positively evaluated several of our design choices, in particular, the aggregation of transactions arcs (see Section 5.1, especially when paired with firm filtering), the guidance on the time selector (see Section 5.2) and the base map visualization (see quotes from Section 7.4). Considering also the distribution and number of insights, and the observed usage patterns on all available scales, we argue that *Sabrina 2.0* achieves **G1**.

We observed that the use of inferred transaction models generated a noticeable increase of insights, especially in the local scale. The missing transaction information in the data encouraged users to explore the transaction models in detail, particularly for companies they had prior interest in, focusing first on a firm's local investments. Once the local investigation was carried out, users activated the transaction bundling feature to uncover new model‐based insights at larger scales as well. The increase in the number of insights due to the supplement of missing information through inferred models suggests that the feature is a valuable addition to the accustomed financial data exploration workflow. Therefore, we argue that **G2** (benefit through model integration) is achieved as well.

Finally, we base our discussion of **G3** (benefit to the experts’ workflow) on the distribution of findings over the two different user groups involved in the evaluation as well as on specific feedback. We already observed that the two groups of users had a ‘mirrored’ distribution of findings, both in terms of the exploration type (with and without the consideration of models) and in terms of the most prominent scale (see Section 7.4 and Figure 9). We consider this to be a potential consequence of the type of tasks the users usually face during their daily work (see also our discussion in Section 7.4). The participants from industry are more interested in the local effects of policies: Most of their observations, in fact, lie in the regional/local scale (17 out of 22). On the other hand, academic researchers focus on simulating and understanding economic phenomena at a larger scale. This was reflected in their insight count skewed towards national/regional scales (24 out of 34). We asked the users whether they would consider using this tool in their daily workflow. Industry participants replied positively, though suggesting to add features for direct manipulation of the models to simulate shock and resilience scenarios, as ‘*[…] with these we could use it all the time*’. One of the participants from academia, after the study session ended, asked to use *Sabrina 2.0* to investigate their own data. This feedback indicates that *Sabrina 2.0* achieves **G3**, suggesting that our system would be beneficial in the participants’ daily workflow. However, three users remarked the complexity of the system, adding that more time would be needed for them to fully explore the data and get comfortable with all features.

## 8 Discussion and Lessons Learned

The results of the evaluation give us a good impression of how experts from different fields approach and use *Sabrina 2.0*, but also highlight its limitations. The concept of the transaction models proved not to be as intuitive as we expected. While the idea of merging different sources of information was received with cautious interest by all of the evaluation participants, the procedure behind the model generation through the combination of logical statements and an SMT solver was hard to grasp for the industry participants—especially because none of them had experience with such methods. Participants from academia had more experience with the simulation of financial environments, albeit using different techniques in their work (i.e. agent‐based modelling, see Section 7.2). A visual constraint builder could potentially help in conveying the idea, and can thus be considered a valuable extension of *Sabrina 2.0* for future work.

Both visual and computational scalability are potential limitations of the system. The transaction arcs are vulnerable to clutter and clipping when the number of elements to display increases. To address the issue, the system offers both filtering and edge aggregation. While aggregation causes a loss of resolution, our evaluation suggests that the benefits of this technique outweigh the potential disadvantages (see Section 7). The computational scalability of *Sabrina 2.0* partially depends on the generation of the models. The method we adopt (see Section 4) utilizes SAT/SMT satisfiability, which is a computationally expensive operation—transaction networks for e.g. 1000 firms may take hours to compute, depending on the complexity of the specified constraints. As such, model generation is intended to be carried out offline. Concerning the run‐time performance of *Sabrina 2.0*, we stress‐tested the system by loading the entire Sabina data base (180k firms). In a local setup (i.e. without download times for the data files), the system took 15 s to load the initial screen, and no more than 5 on data processing operations such as metric recalculations, firm re‐binning and model parsing times. Exploration operations were always performed quickly enough to support interactivity. Scaling to larger datasets might require adopting a client–server architecture with a dedicated database for data storage and processing.

With the experience gathered from this and our previous work [ATJ*19], we now crystallize the most important lessons learned. While we did not specifically investigate in our evaluation the impact of the 3D approach on the user experience, we found our participants to be sure‐footed and engaged when using our system, despite being used to tabular views and numeric computing software in their daily workflow. This could be partly due to the increased popularity of 3D interfaces and visualizations, with some notable examples especially in cartography applications (e.g. Google Earth [Gooa] and 3D features of Maps [Goob]). Therefore, the third dimension can be considered as a suitable extension to 2D map‐based approaches when additional dimensions are required for data encoding, while keeping the underlying geographical context. Guidance [CGM*17] should be considered as an important principle when designing a VA system. Using user interface elements to actively aid users in their analysis process, e.g. by changing the colour of an element to suggest a trend on a slider, improves their experience with the system significantly. Finally, we found that support for incorporating domain knowledge of a user, especially within the context of visualizing incomplete or personalized data, is a well‐received but yet under‐explored feature—enabling a user to be more involved in the analysis process, potentially unlocking deeper insights.

## 9 Conclusion and Outlook

In this paper, we presented *Sabrina 2.0*, a VA approach for the analysis of micro‐ and macroscopic economic data that integrates a pipeline to infer firm‐to‐firm transaction networks. We designed our system with the help of domain experts to further improve and extend our preliminary work [ATJ*19]. We evaluated our system with a qualitative insight‐based evaluation that helped us to identify the system's strong and weak points. The evaluation gave us encouraging results, as the feedback validated our design choices, indicating that our design goals were reached.

The natural evolution of *Sabrina 2.0* would be the integration of a visual tool for the generation of financial transaction models, for instance through a domain‐specific language. While generating large transaction networks is computationally expensive, the idea can still be achieved in a ‘*model*‐as‐a‐service’ fashion. Since the system is built to accept any type of compatible model encoded in JSON (see Section 5), the use of transaction networks that were generated using different methods is already supported. Interesting future avenues also include the expansion of *Sabrina 2.0* to include multiple national economies, as well as introducing models and constraints that also consider import/export of goods and services between countries. Such features would provide an important step in improving our understanding of the relationship between global and local economies.

## Supporting information

Supplemental Video S1

## References

[cgf14723-bib-0001] [AA13] Andrienko N. , Andrienko G. : Visual analytics of movement: An overview of methods, tools and procedures. Information Visualization 12, 1 (2013), 3–24.

[cgf14723-bib-0002] [ATJ*19] Arleo A. , Tsigkanos C. , Jia C. , Leite R. A. , Murturi I. , Klaffenböck M. , Dustdar S. , Wimmer M. , Miksch S. , Sorger J. : Sabrina: Modeling and visualization of financial data over time with incremental domain knowledge. In Proceedings of the 2019 IEEE Visualization Conference (VIS) (2019), IEEE, pp. 51–55.

[cgf14723-bib-0003] [BBBL11] Boyandin I. , Bertini E. , Bak P. , Lalanne D. : Flowstrates: An approach for visual exploration of temporal origin‐destination data. Computer Graphics Forum 30, 3 (2011), 971–980.

[cgf14723-bib-0004] [BOB07] Birch C. P. , Oom S. P. , Beecham J. A. : Rectangular and hexagonal grids used for observation, experiment and simulation in ecology. Ecological Modelling 206, 3‐4 (2007), 347–359.

[cgf14723-bib-0005] [BSF17] Battersby S. E. , Strebe D. d. , Finn M. P. : Shapes on a plane: Evaluating the impact of projection distortion on spatial binning. Cartography and Geographic Information Science 44, 5 (2017), 410–421.

[cgf14723-bib-0006] [BT18] Barrett C. , Tinelli C. : Satisfiability modulo theories. In Handbook of Model Checking. Springer, Cham, Switzerland (2018), pp. 305–343.

[cgf14723-bib-0007] [Car90] Carr D. B. : Looking at Large Data Sets Using Binned Data Plots. Tech. Rep., Pacific Northwest Lab., Richland, WA, USA, 1990.

[cgf14723-bib-0008] [CDE*06] Chevaleyre Y. , Dunne P. E. , Endriss U. , Lang J. , Lemaitre M. , Maudet N. , Padget J. , Phelps S. , Rodriguez‐Aguilar J. A. , Sousa P. : Issues in multiagent resource allocation. Informatica 30, 1 (2006), 3–31.

[cgf14723-bib-0009] [CGK*07] Chang R. , Ghoniem M. , Kosara R. , Ribarsky W. , Yang J. , Suma E. , Ziemkiewicz C. , Kern D. , Sudjianto A. : Wirevis: Visualization of categorical, time‐varying data from financial transactions. In Proceedings of the IEEE Symposium on Visual Analytics Science and Technology (2007).

[cgf14723-bib-0010] [CGM*17] Ceneda D. , Gschwandtner T. , May T. , Miksch S. , Schulz H. , Streit M. , Tominski C. : Characterizing guidance in visual analytics. IEEE Transactions on Visualization and Computer Graphics 23, 1 (2017), 111–120. 27514054 10.1109/TVCG.2016.2598468

[cgf14723-bib-0011] [Cha02] Charemza W. W. : Guesstimation. Journal of Forecasting 21, 6 (2002), 417–433.

[cgf14723-bib-0012] [CLNL87] Carr D. B. , Littlefield R. J. , Nicholson W. , Littlefield J. : Scatterplot matrix techniques for large n. Journal of the American Statistical Association 82, 398 (1987), 424–436.

[cgf14723-bib-0013] [COW92] Carr D. B. , Olsen A. R. , White D. : Hexagon mosaic maps for display of univariate and bivariate geographical data. Cartography and Geographic Information Systems 19(1992), 228–236.

[cgf14723-bib-0014] [CPMP98] Coppola D. M. , Purves H. R. , McCoy A. N. , Purves D. : The distribution of oriented contours in the real world. Proceedings of the National Academy of Sciences 95, 7 (1998), 4002–4006. 10.1073/pnas.95.7.4002 PMC19952 9520482

[cgf14723-bib-0015] [DGL*18] Didimo W. , Giamminonni L. , Liotta G. , Montecchiani F. , Pagliuca D. : A visual analytics system to support tax evasion discovery. Decision Support Systems 110(2018), 71–83.

[cgf14723-bib-0016] [DMB08] De Moura L. , Bjørner N. : Z3: An efficient smt solver. In Proceedings of the International Conference on Tools and Algorithms for the Construction and Analysis of Systems (2008).

[cgf14723-bib-0017] [DML14] Dumas M. , McGuffin M. J. , Lemieux V. L. : Financevis.net—a visual survey of financial data visualizations. In Poster and Extended Abstract (Nov. 2014).

[cgf14723-bib-0018] [DWCM18] Dong W. , Wang S. , Chen Y. , Meng L. : Using eye tracking to evaluate the usability of flow maps. ISPRS International Journal of Geo‐Information 7, 7 (2018), 281.

[cgf14723-bib-0019] [FLVW16] Flood M. D. , Lemieux V. L. , Varga M. , Wong B. W. : The application of visual analytics to financial stability monitoring. Journal of financial stability 27(2016), 180–197.

[cgf14723-bib-0020] [Git] Git : Sabrina 2.0 github repository. https://github.com/EngAAlex/Sabrina‐2.0. Accessed: 2022‐11‐18.

[cgf14723-bib-0021] [Gooa] Google : Earth. https://earth.google.com/. Accessed: 2022‐04‐07.

[cgf14723-bib-0022] [Goob] Google : Maps. https://maps.google.com/. Accessed: 2022‐04‐07.

[cgf14723-bib-0023] [HB03] Harrower M. , Brewer C. A. : Colorbrewer.org: An online tool for selecting colour schemes for maps. The Cartographic Journal 40, 1 (2003), 27–37.

[cgf14723-bib-0024] [Her16] Herbain C. A. : Towards a single eu vat area. British Tax Review, 4 (2016), 402–407.

[cgf14723-bib-0025] [JSM*18] Jenny B. , Stephen D. M. , Muehlenhaus I. , Marston B. E. , Sharma R. , Zhang E. , Jenny H. : Design principles for origin‐destination flow maps. Cartography and Geographic Information Science 45, 1 (2018), 62–75.

[cgf14723-bib-0026] [KC19] Lugmayr A., Lim Y., Hollick J., Khuu J. , Chan F. : Financial data visualization in 3d on immersive virtual reality displays. Enterprise Applications, Markets and Services in the Finance Industry (2019), 118–130.

[cgf14723-bib-0027] [KCA*16] Ko S. , Cho I. , Afzal S. , Yau C. , Chae J. , Malik A. , Beck K. , Jang Y. , Ribarsky W. , Ebert D. S. : A survey on visual analysis approaches for financial data. Conputer Graphics Forum 35, 3 (2016), 599–617.

[cgf14723-bib-0028] [KMJE12] Ko S. , Maciejewski R. , Jang Y. , Ebert D. S. : MarketAnalyzer: An interactive visual analytics system for analyzing competitive advantage using point of sale data. Computer Graphics Forum 31, 3 (2012), 1245–1254.

[cgf14723-bib-0029] [KMU] KMU : Wirtschaftskraft KMU. https://news.wko.at/news/oesterreich/Wirtschaftskraft_KMU.html. Accessed: 2021‐09‐21.

[cgf14723-bib-0030] [KSH*99] Kirkland J. D. , Senator T. E. , Hayden J. J. , Dybala T. , Goldberg H. G. , Shyr P. : The NASD regulation advanced‐detection system (ADS). AI Magazine 20, 1 (1999), 55.

[cgf14723-bib-0031] [LAS*20] Leite R. A. , Arleo A. , Sorger J. , Gschwandtner T. , Miksch S. : Hermes: Guidance‐enriched visual analytics for economic network exploration. Visual Informatics 4, 4 (2020), 11–22.

[cgf14723-bib-0032] [LGM*18] Leite R. A. , Gschwandtner T. , Miksch S. , Kriglstein S. , Pohl M. , Gstrein E. , Kuntner J. : EVA: Visual analytics to identify fraudulent events. IEEE Transactions on Visualization and Computer Graphics 24, 1 (2018), 330–339. 28880181 10.1109/TVCG.2017.2744758

[cgf14723-bib-0033] [MA14] Miksch S. , Aigner W. : A matter of time: Applying a data–users–tasks design triangle to visual analytics of time‐oriented data. Computers & Graphics 38(2014), 286–290.

[cgf14723-bib-0034] [MGBC07] Mirel B. , Goldsmith P. , Brath R. , Cort B. : Visual analytics for model‐based policy analysis: Exploring rapid changes in commodities markets. In Proceedings of the 8th Annual International Conference on Digital Government Research, Bridging Disciplines & Domains (2007).

[cgf14723-bib-0035] [Mun09] Munzner T. : A nested model for visualization design and validation. IEEE Transactions on Visualization and Computer Graphics 15, 6 (2009), 921–928. 19834155 10.1109/TVCG.2009.111

[cgf14723-bib-0036] [Nor06] North C. : Toward measuring visualization insight. IEEE Computer Graphics and Applications 26, 3 (2006), 6–9. 10.1109/mcg.2006.70 16711210

[cgf14723-bib-0037] [PMT17] Poledna S. , Miess M. , Thurner S. : Economic Forecasting with an Agent‐based Model. Tech. Rep, 2017.

[cgf14723-bib-0038] [Sab] Sabina : Wirtschaftsuniversität wien: Sabina ‐ info ‐ datenbanken. https://www.wu.ac.at/bibliothek/recherche/datenbanken/info/sabina/. Accessed: 2022‐11‐18.

[cgf14723-bib-0039] [SAHCV20] Schroeder K. , Ajdadilish B. , Henkel A. P. , Calero Valdez A. : Evaluation of a financial portfolio visualization using computer displays and mixed reality devices with domain experts. In Proceedings of the 2020 CHI Conference on Human Factors in Computing Systems (2020), pp. 1–9.

[cgf14723-bib-0040] [SND05] Saraiya P. , North C. , Duca K. : An insight‐based methodology for evaluating bioinformatics visualizations. IEEE Transactions on Visualization and Computer Graphics 11, 4 (2005), 443–456. 16138554 10.1109/TVCG.2005.53

[cgf14723-bib-0041] [TASD19] Tsigkanos C. , Arleo A. , Sorger J. , Dustdar S. : How do firms transact? Guesstimation and validation of financial transaction networks with satisfiability. In Proceedings of the 20th International Conference on Information Reuse and Integration for Data Science (IRI) (2019), pp. 15–22.

[cgf14723-bib-0042] [TDL*15] Timmer M. P. , Dietzenbacher E. , Los B. , Stehrer R. , de Vries G. J. : An illustrated user guide to the world input–output database: The case of global automotive production. Review of International Economics 23, 3 (2015), 575–605.

[cgf14723-bib-0043] [Teg99] Tegarden D. P. : Business information visualization. Communications of the Association for Information Systems 1, 1 (1999), 4.

[cgf14723-bib-0044] [TK08] Tekušová T. , Kohlhammer J. : Visual analysis and exploration of complex corporate shareholder networks. Visualization and Data Analysis 6809 (2008), 152–161.

[cgf14723-bib-0045] [TR10] Ten Raa T. : Input‐Output Economics: Theory and Applications: Featuring Asian Economies. World Scientific, Singapore, 2010.

[cgf14723-bib-0046] [VFAA17] Vrotsou K. , Fuchs G. , Andrienko N. , Andrienko G. : C. Journal of Geovisualization and Spatial Analysis 1, 1‐2 (2017), 1.

[cgf14723-bib-0047] [WA08] Weinstein L. , Adam J. A. : Guesstimation: Solving the world's problems on the back of a cocktail napkin. American Journal of Physics 76(2008), 887.

[cgf14723-bib-0048] [Wan19] Wang Y. : Deck.gl: Large‐scale web‐based visual analytics made easy. IEEE Workshop on Visualization in Practice (2017).

[cgf14723-bib-0049] [YDJ*18] Yang Y. , Dwyer T. , Jenny B. , Marriott K. , Cordeil M. , Chen H. : Origin‐destination flow maps in immersive environments. IEEE Transactions on Visualization and Computer Graphics 25, 1 (2018), 693–703. 10.1109/TVCG.2018.2865192 30136995

